# Radiation treatment planning study to investigate feasibility of delivering Immunotherapy in Combination with Ablative Radiosurgery to Ultra‐High DoSes (ICARUS)

**DOI:** 10.1002/acm2.13204

**Published:** 2021-02-24

**Authors:** Michelle B. Rokni, Kelli B. Pointer, Jonathan George, Jason J. Luke, Steven J. Chmura, Gage Redler

**Affiliations:** ^1^ Department of Radiation and Cellular Oncology The University of Chicago Medicine Chicago IL USA; ^2^ Department of Medicine Division of Hematology/Oncology University of Pittsburgh and UPMC Hillman Cancer Center Pittsburgh PA USA; ^3^ Department of Radiation Oncology Moffitt Cancer Center Tampa FL USA

**Keywords:** immunotherapy, SBRT, treatment planning, ultra‐high dose

## Abstract

**Purpose:**

Immune checkpoint inhibitors improve survival in metastatic diseases for some cancers. Multisite SBRT with pembrolizumab (SBRT + Pembro) was shown to be safe with promising local control using biologically effective doses (BEDs) = 95–120 Gy. Increased BED may improve response rate; however, SBRT doses are limited by surrounding organs at risk (OARs). The purpose of this work was to develop and validate methods for safe delivery of ultra‐high doses of radiation (BED_10_ > 300) to be used in future clinical trials.

**Methods and Materials:**

The radiation plans from 15 patients enrolled on a phase I trial of SBRT + pembro were reanalyzed. Metastatic disease sites included liver (8/15), inguinal region (1/15), pelvis (2/15), lung (1/15), abdomen (1/15), spleen (1/15), and groin (1/15). Gross tumor volumes (GTVs) ranged from 80 to 708 cc. Following the same methodology used in the Phase I trial on which these patients were treated, GTVs > 65 cc were contracted to a 65 cc subvolume (SubGTV) resulting in only a portion of the GTV receiving prescription dose. Volumetric modulated arc therapy (VMAT) was used to plan treatments BED_10_ = 360 Gy. Plans utilizing both 6FFF and 10FFF beams were compared to clinical plans delivering BED_10_ = 112.50 Gy. The target primary goal was V100% > 95% with a secondary goal of V70% > 99% and OAR objectives per the trial. To demonstrate feasibility, plans were delivered to a diode array phantom and evaluated for fidelity using gamma analysis.

**Results:**

All 30 plans met the secondary coverage goal and satisfied all OAR constraints. The primary goal was achieved in 12/15 of the 6FFF plans and 13/15 of the 10FFF plans. Average gamma analysis passing rate using criteria of 3% dose difference and 3, 2, and 1 mm were 99.1 ± 1.0%, 98.5 ± 1.6%, and 95.1 ± 3.8%, respectively.

**Conclusion:**

Novel VMAT planning approaches with clinical treatment planning software and linear accelerators prove capable of delivering radiation doses in excess of 360 Gy BED_10_ to tumor subvolumes, while maintaining safe OAR doses.

## 1 INTRODUCTION

Cancer immunotherapy, especially anti‐programmed death receptor 1 (PD1) antibody treatment, is associated with greater efficacy in patients with tumors harboring higher levels of tumor infiltrating lymphocytes and T‐cell‐inflamed gene expression.^1^, ^2^ Stereotactic body radiation therapy (SBRT) has been shown to activate the innate and adaptive immune response.^3^ These data together suggest that SBRT may improve tumor control in combination with immunotherapy with several reported trials offering early support for this concept.^4^, ^5^, ^6^

Our group previously reported the initial results of a phase I clinical trial investigating the safety of treating patients with metastatic disease in multiple sites with SBRT followed by pembrolizumab.^4^ Grade 3 toxicities were <10% with combined therapy and the irradiated tumor control rate was observed as 89.5% at 12 months, with 15% of those tumors receiving only a portion of the prescribed radiation dose. The trial implemented a 65 cc treatment volume limit with intent to avoid normal tissue toxicity.^7^ Other groups have employed similar partial treatment studies with similar results.^8^

While the local control rate approaches 90% at 12 months after treatment with SBRT and immunotherapy, one strategy to continue to improve outcomes and potentially increase the immune response would be to further escalate the SBRT dose. The overall tumor control in our previous study was achieved using a biologically effective dose (BED) of roughly 100 Gy. Various studies have demonstrated that higher BED rates lead to better local control^9^, ^10^, ^11^ with long‐term follow‐up. However, while a higher BED provides better local control, radiation doses are typically limited by dosimetric constraints of surrounding organs at risk (OARs). SBRT enables highly effective treatments of radiation with a rapid dose falloff enabling increased tumor dose while sparing surrounding OARs. Based on our previous study demonstrating that local control is similar in tumors that receive partial or full irradiation,^7^ we hypothesized that by only partially irradiating the tumors we could deliver higher doses of radiation, while still meeting OAR constraints that typically limit radiation doses. This increase in BED, in turn, has the potential to improve response rate.

This paper describes our novel planning approach, using commercially available FDA approved tools (Eclipse treatment planning system (TPS) and TrueBeam Linac [Varian Medical Systems, Palo Alto, CA)], to deliver immunotherapy in combination with ablative radiosurgery to ultra‐high doses (ICARUS). Multiple treatment beam energies (with applicability depending on tumor location), increased numbers of treatment arcs [for efficient use of increased monitor unit (MU) requirement and expanded optimization solution space], and additional planning structures/objectives (to enforce dose buildup within the subvolumes (SubGTV) that is spatially optimal for OAR sparing) were all implemented. With this planning approach, we were able to create ICARUS plans, while still meeting OAR dose constraints validated for safety with a phase I clinical trial, therefore achieving comparable isotoxicity. These plans were used to treat a clinical quality assurance phantom to demonstrate deliverability.

## 2 MATERIALS AND METHODS

### 2.A Patient and tumor eligibility criteria

The first 15 patients who were previously enrolled on an institutional phase I trial that investigated the safety of SBRT + pembro (NCT02608385), and were not excluded due to criteria discussed below, were chosen to be included in this retrospective study. Only large lesions (>65 cc) that had SubGTVs and were treated as single‐target, single‐isocenter were used for this study. In addition, lesions that did not meet the clinical trial primary coverage goal when 45 Gy was prescribed on the clinical trial protocol were excluded.

Disease sites included liver (8/15), pelvis (2/15), inguinal nodes (1/15), spleen (1/15), lung (1/15), abdomen (1/15), and groin (1/15). A list of the disease site planned for each patient is shown in Table 1. Based on the patient selection criteria discussed above, liver lesions were more applicable for this study, as they are typically larger (therefore, a SubGTV is needed), and there are less nearby OARs (therefore more clinical plans passed the primary clinical trial target coverage goal). In addition to the eight liver lesions, a wide variety of other disease sites were sampled to show the general applicability of this method.

**TABLE 1:** Tumor coverage for the 6 FFF photon and 10 FFF photon plans.

Patient	Disease site	6 FFF coverage		10FFF coverage	
		Primary goal (V_100%_) [%]	Secondary goal (V_70%_) [%]	Primary goal (V_100%_) [%]	Secondary goal (V_70%_) [%]
1	Rt Inguinal Node	55.4^a^	100	92.1^a^	100
2	Pelvis	97.3	100	99.5	100
3	Liver	99.9	100	99.9	100
4	Liver	96.5	100	89^a^	99.4
5	Liver	99.9	100	99.9	100
6	Liver	95.3	100	95.5	100
7	Liver	100	100	100	100
8	Spleen	88+	100	97.4	100
9	Liver	95	100	95.5	100
10	Pelvis	98.6	100	100	100
11	Lung	100	100	100	100
12	Abdomen	41^a^	100	99.7	100
13	Liver	99	100	99.6	100
14	Groin	96	100	99.64	100
15	Liver	96.4	100	98.9	100
Avg	–	97.8 ± 1.9	100 ± 0	98.9 ± 1.7	100 ± 0.2

^a^Plan did not pass the coverage goal (Primary: V_100%_ > 95%; Secondary: V_70%_ > 99%).

### 2.B SBRT dosing

**FIG. 1:**
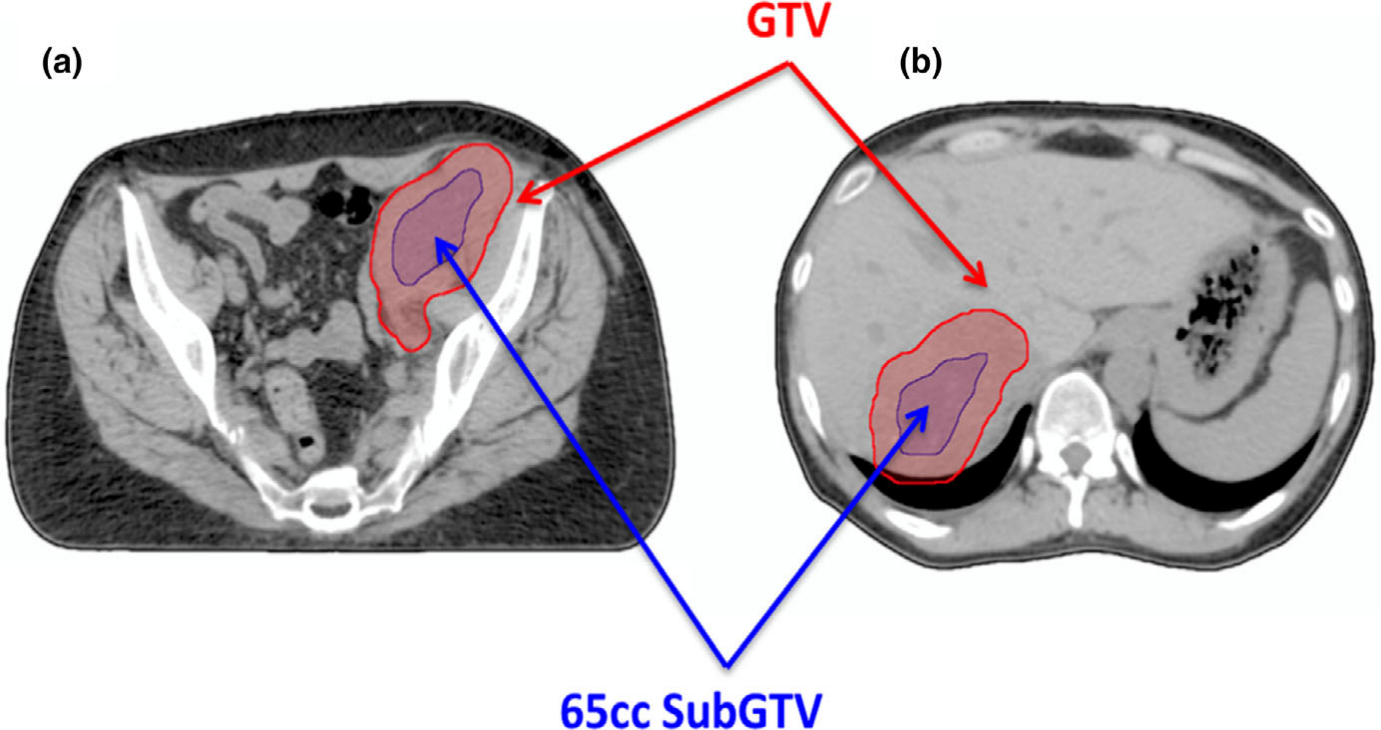
Contracted 65cc planning SubGTV (blue) and contoured GTV volumes (red) for (a) pelvis and (b) liver patients.

In the trial, each lesion received SBRT to a dose of 30–50 Gy over three to five fractions. The trial required that the prescription dose be delivered to a target with a maximum volume of 65 cc. A 65 cc subvolume was chosen in the initial clinical trial based on consensus for multisite SBRT establishing a target size limited to 5 cm diameters (i.e., a 65 cc sphere).^12^ Therefore, gross tumor volumes (GTVs) >65cc were contracted to a ~65 cc SubGTV and received the prescription dose to only this part of the tumor. This contraction was geometrically based rather than biologically based (i.e., contractions were generally uniform). The GTV and OAR contours used in the original SBRT plans were also used for this ICARUS planning study. Examples of this GTV contraction are shown in Fig. 1.

### 2.C Treatment planning approach

Standard approaches to volumetric modulated arc therapy (VMAT) planning were modified in order to force the clinical TPS to optimize ICARUS plans with both a higher dynamic range of doses and higher spatial dose gradients than currently used in clinical practice. To help create the desired dose distribution, a minimum MU limit of ~11 000 MU was used to force higher doses, while a maximum MU limit of ~13 000 MU was used to limit modulation in the optimizer. These values were determined empirically from initial efforts to create these ICARUS plans within the TPS. Additionally, an automatic normal tissue objective (NTO) with a weight of ~150 was utilized. Four ring structures external to the SubGTV (0–5 mm, 5–10 mm, 10–20 mm, and 20–50 mm) were made to assist with creating a steeper dose falloff outside of the target, and five to seven internal concentric 1 mm ring structures were used to help control dose buildup and centralize hot spots within the target. All ICARUS plans were created using the Eclipse TPS and calculated using AcurosXB v11. All treatments were planned for a TrueBeam linear accelerator equipped with HD‐MLC and calculated with a 2.0 mm dose grid size. VMAT was used to create the treatment plans, prescribing 90 Gy in three fractions to the 65cc target SubGTV. Both the original treatment plans and ICARUS plans were created by physicists at the same institution, but in both cases, these were not necessarily all by the same individual. However, all plans were reviewed by the same radiation oncologist, the principal investigator of the Phase I trial.

For each patient, two different treatment plans were created using different beam energies. The first plan used 6 MV flattening filter free (6FFF) photons. This is generally a more commonly used beam energy for VMAT SBRT treatments and is what was used for all clinical trial treatments. The second plan utilized 10 MV flattening filter free (10FFF) photons. This beam energy allows for higher dose rate (2400 vs 1400 MU/min), as well as higher energy which may provide better skin sparing, but alternatively there is a greater (albeit relatively small) unknown neutron contamination for this energy. Each plan consisted of six to eight partial/full arcs, in contrast to the two to four arcs that were typically used in the clinical trial plans. Examples of typical gantry and collimator angles used for the ICARUS study are shown in Fig. S1.

All plans had a SubGTV coverage primary goal of V100% > 95% and a secondary goal of V70% > 99%. Following the trial protocol, there were no coverage goals for the full (>65 cc) GTV. OAR objectives followed the trial protocol^4^ and are shown in Table S1. These OAR tolerances have been demonstrated to be safe for patients receiving immunotherapy and SBRT and therefore were considered hard constraints.^7^ In addition to the protocol OAR constraints, additional conservative bone constraints of V50 Gy < 0.03 cc and V40 Gy < 5 cc were used (i.e., applying clinical protocol constraint for rib fracture avoidance to all bony anatomy). Since the purpose of this study was to determine if high doses of radiation could be safely planned and delivered to patients while still respecting OAR tolerances, the planning approach prioritized OAR tolerances, while trying to achieve optimal target coverage.

### 2.D Plan quality metrics

After the optimization of each plan, dose volume histograms (DVHs) were generated and evaluated following the dosimetric criteria of the trial protocol for the SubGTV and nearby OARs. Additionally, a wide variety of commonly used planning metrics for assessing clinical acceptability in other clinical protocols/trials were chosen to further evaluate these plans. These planning metrics included: modulation factor (MF), conformity index (CI), high dose spillage metric (R_105%_), low/intermediate dose spillage volume/magnitude metric (R_50%_), gradient index (GI), homogeneity index (HI), and low/intermediate dose spillage location metric (D2 cm).

Modulation factor is defined as

(1) $$MF=\frac{\mathrm{MU}}{D_{\mathrm{Rx}}},$$

where *MU* is the number of monitor units in a given plan and *D_Rx_* is the prescription dose in cGy. This metric was chosen to evaluate plan delivery efficiency and avoid unrealistic optimization results. The goal in this work was to keep this below a value of 4, with a value less than 5 considered to be acceptable. These values are based on departmental suggested guidelines for consistency when moving to higher doses with respect to clinical trial plans and in order to restrict contributions from poorly modeled MLC leakage and scatter dose contribution, which has been shown to be proportional to plan MU. Increased MF has also been shown to correlate with increased plan complexity and increased patient‐specific quality assurance gamma failing rates. While there are not necessarily definitive guidelines for acceptable MF values, data suggest that MF >~3.5–4.0 tends to be where these secondary dose contributions reach more significance relative to the primary dose contribution and gamma pass rates subsequently decrease.^13^, ^14^

Conformity index is defined as

(2) $$CI=\frac{V_{isodose\_100\%}}{V_{\mathrm{SubGTV}}},$$

where *V_isodose, 100%_* is the volume enclosed by the prescription isodose line and *V_SubGTV_* is the volume of the SubGTV. The *CI* quantifies high dose spillage magnitude, but not location, by measuring how well a planned prescription isodose line conforms to the target.^15^ A value of 1.0 is optimal, values < 1.0 indicate undercoverage, and values > 1.0 represent lack of conformity. Lack of conformality spreads the high dose beyond the target (assuming that the two volumes defined in Eq. (2) are in the same physical location).

R_105%_ is defined as

(3) $$R_{105\%\;}=\frac{V_{isodose\_105\%}}{V_{\mathrm{SubGTV}}},$$

where *V_isodose, 105%_* is the volume of the 105% isodose line outside of the SubGTV. This metric is related to the high dose spillage location, not magnitude, as a complement to the *CI*. As per RTOG0915, *R_105%_* should be <0.15.^16^, ^17^

R_50%_ is defined as

(4) $$R_{50\%\;}=\frac{V_{isodose\_50\%}}{V_{\mathrm{SubGTV}}}.$$

This metric is commonly used in clinical trials utilizing SBRT as means of quantifying the low to intermediate dose spillage magnitude, but not location.^16^, ^17^ As per RTOG 0915, for a 65cc target, *R_50%_* should be <3.63, with a value <4.85 representing only a minor deviation from protocol.^16^

The low to intermediate dose spillage location metric (*D_2cm_*) is defined as the maximum dose at a distance of 2 cm from the target, as used in numerous SBRT trial protocols.^16^, ^17^ As per RTOG0915, for a 65 cc target, *D_2cm_* should be <65% of the prescription, with a value <83.75% representing only a minor deviation from protocol.^16^

Gradient index is defined as

(5) $$GI=\frac{V_{isodose\_50\%}}{V_{isodose\_100\%}},$$

where *V_isodose, 50%_* is the volume enclosed by the 50% isodose line.^18^ This is a commonly used metric in SBRT to quantify how rapidly dose falls off when moving away from the target, assuming that the prescription isodose line is made to be as conformal to the target as possible. While there is no definitive value that must be achieved here, in a study with 90 lung SBRT patients, the GI that was found to be acceptable was 4.20 ± 0.60.^19^

Homogeneity index is defined as

(6) $$HI=\frac{D_{\max}}{D_{\mathrm{Rx}}},$$

where *D_max_* is the maximum dose. This metric quantifies hot spot magnitude. The HI for a target that has perfect homogeneity coverage by the prescription dose will be 1.0. As per RTOG0915, *D_Rx_* should be between 60 and 90% of *D_max_*, implying that *HI* should be between 1.11 and 1.67.^16^

### 2.E Phantom measurements

To demonstrate the deliverability of the plans in this study, patient plans were delivered to an ArcCHECK quality assurance phantom (Model 1220, Sun Nuclear, Melbourne, FL), which is currently used clinically at our institution. The ArcCHECK phantom is a cylindrical phantom containing 1386 helically wrapped diode dosimeters with 1 mm spacing for detection of spatial dose distributions. Measured dose was compared to a computed dose distribution calculated on the ArcCHECK phantom by the Eclipse TPS (2 mm isotropic dose grid). Using the associated SNC software (version 8.4.0.55), the data were analyzed using our clinical global gamma passing rate criteria of 3% dose difference (based on a reasonable expectation of precision for these diode detectors) at 3, 2, and 1 mm distance to agreement with a 10% low‐dose threshold to evaluate each plan. Diode readout occurs every 50 ms, thereby, avoiding issues with dose saturation for these ultra‐high dose plan measurements.

## 3 RESULTS

**FIG. 2:**
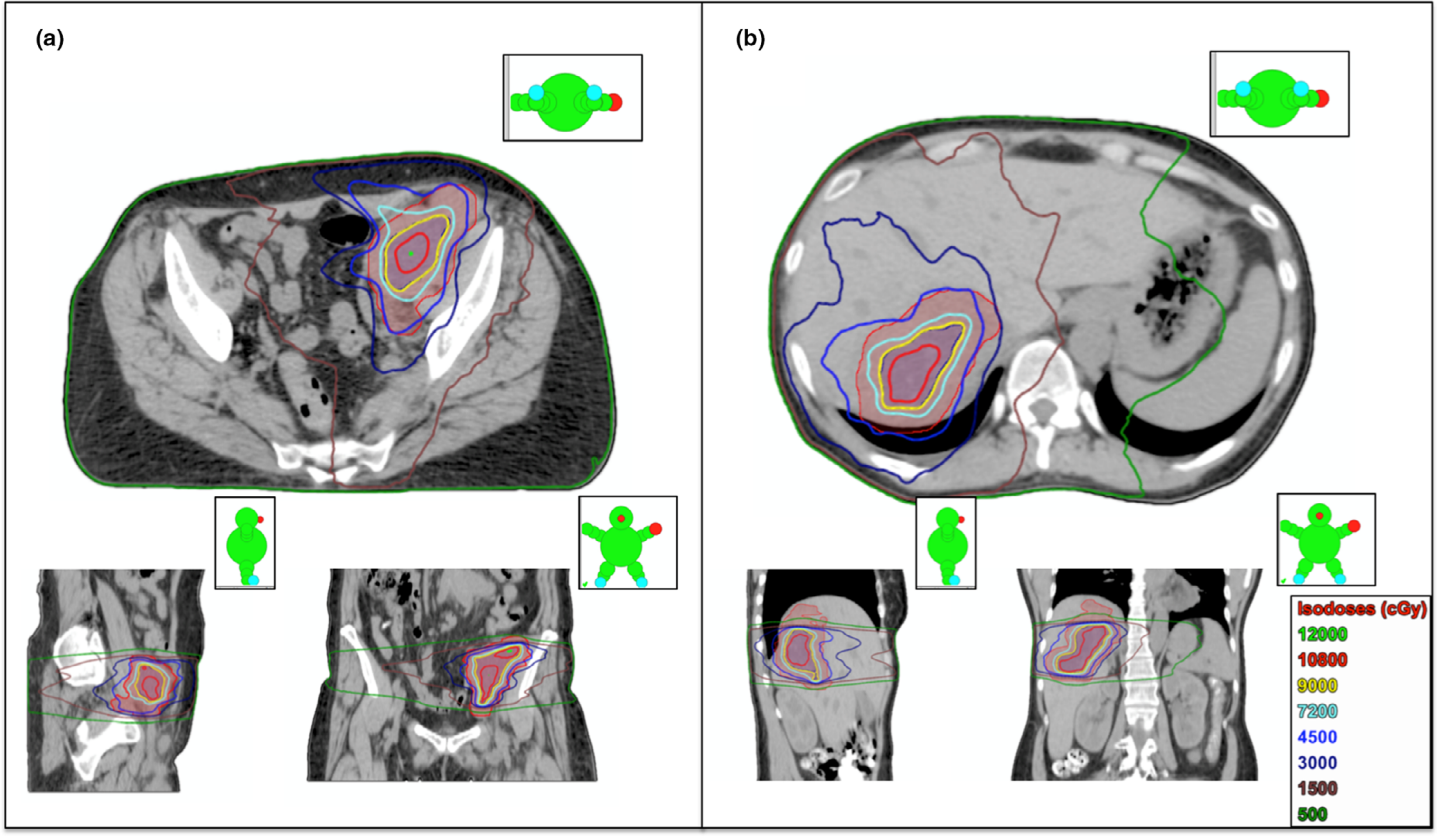
Dose distributions for 6FFF ICARUS plans with a prescribed dose of 90 Gy in three fractions to the 65 cc SubGTV (shaded blue) in three fractions for (a) pelvis patient 02 and (b) liver patient 03.

**FIG. 3:**
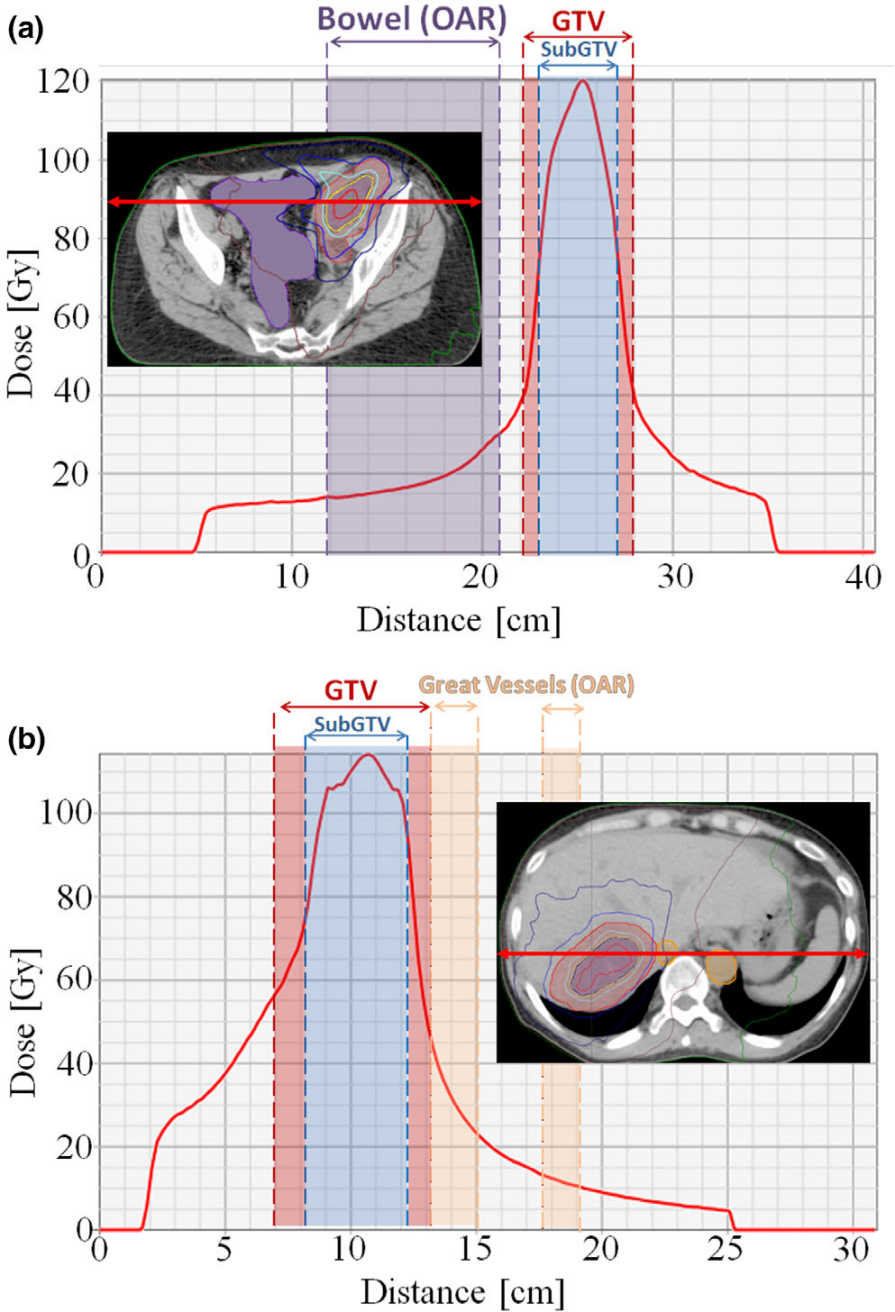
Dose profiles for 6FFF ICARUS plans with a prescribed dose of 90 Gy in three fractions to the 65 cc SubGTV in three fractions for (a) pelvis patient 02 and (b) liver patient 03.

**FIG. 4:**
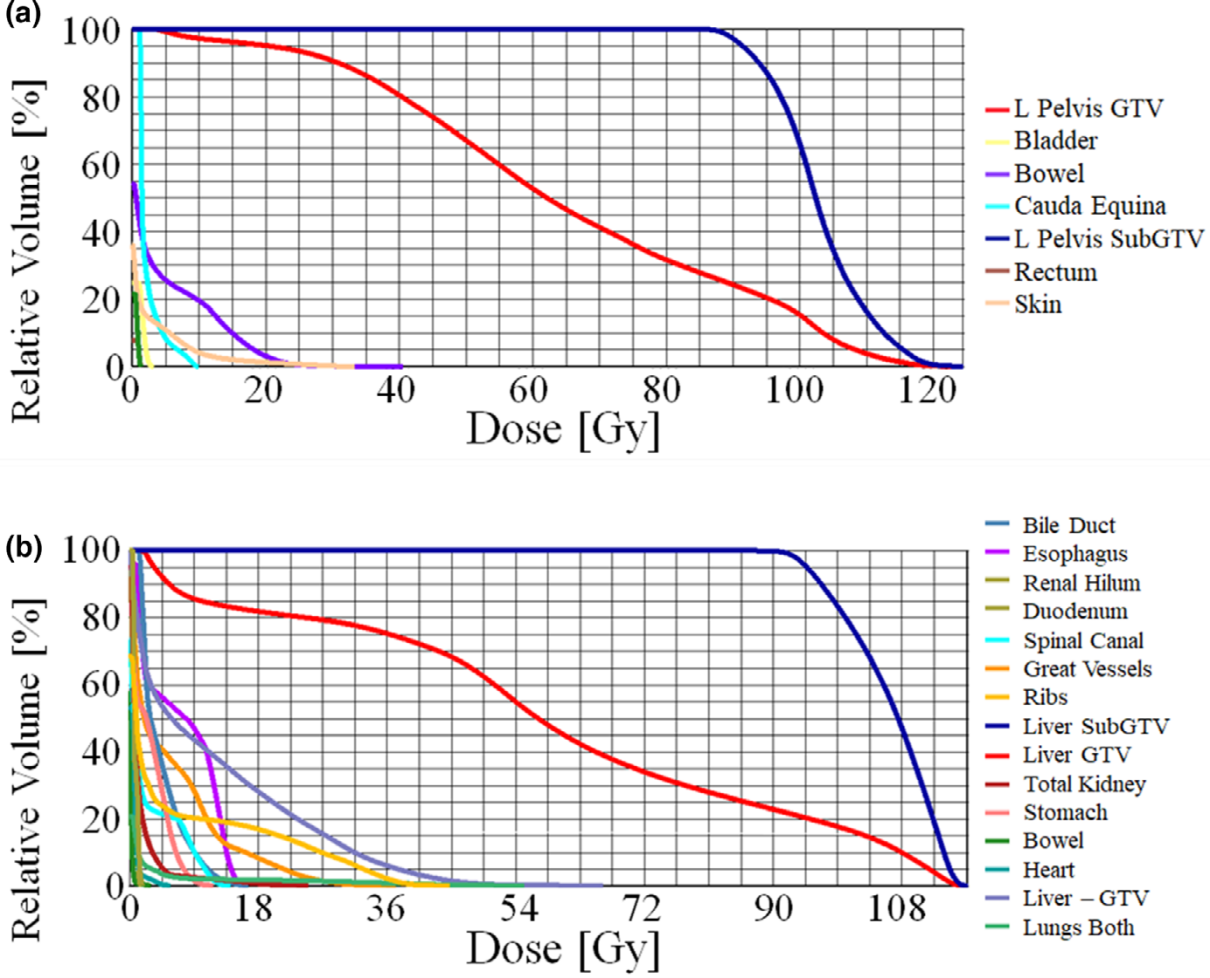
DVHs for 6FFF ICARUS plans for (a) pelvis patient 02 and (b) liver patient 03. The dark blue line represents SubGTV coverage and the red line represents the GTV coverage.

With the use of the planning techniques mentioned above, dose falloff was generally steep enough to deliver ultra‐high doses to the 65 cc SubGTV, while still respecting OAR tolerances. Example dose distributions of the 6FFF ICARUS plans for both a pelvis and liver patient (patients 2 and 5, respectively) are shown in Figs. 2(a) and 2(b), with corresponding dose profiles depicting steep dose falloff in Figs. 3(a) and 3(b). The DVHs for these two plans, shown in Figs. 4(a) and 4(b), again show the drastic difference in doses delivered to the SubGTV vs OARs.

All 30 plans met the secondary coverage goal of V70% > 99%. The primary goal of V100% > 95% was achieved for 12/15 of the 6FFF plans and 13/15 of the 10FFF plans. The primary and secondary coverages for each plan are shown in Table 1. Plans not passing the coverage goal are indicated with an asterisk. Additionally, all OAR constraints were met for the 30 plans. The average maximum dose within the SubGTV was 115.3 Gy (BED_10_ = 558.7 Gy) and 116.8 Gy (BED_10_ = 571.3 Gy) for the 6FFF and 10FFF plans, respectively. Therefore, not only is a significant portion of the 65 cc SubGTV receiving a BED_10_ = 360 Gy, but smaller, more central portions of the tumor receive almost double this dose. This higher BED could, in theory, increase the immune response further, leading to better clinical outcomes. The maximum dose and corresponding BED_10_ values for each plan are shown in Tables 2 and 3.

**TABLE 2:** The calculated planning metrics for each of the 6FFF ICARUS plans.

Patient	D_max_ (Gy)	BED_10_ (Gy)	MF	6FFF					
				CI	GI	HI	R50	R105	D2 cm
1	112.2	532.1	1.78	0.55^a^	4.52^a^	1.25^a^	2.47^a^	0.00^a^	53.2%^a^
2	124.6	642.1	4.54	1.05	3.97	1.38	4.16	0.00	83.4%
3	126.0	655.2	4.09	1.19	4.33	1.40	5.16	0.03	73.1%
4	128.8	682.2	2.65	1.12	3.74	1.43	4.19	0.04	67.1%
5	117.3	576.1	3.17	1.07	3.49	1.30	3.74	0.00	51.8%
6	115.4	558.9	1.89	0.96	3.57	1.28	3.44	0.00	68.0%
7	116.4	568.3	4.44	1.13	3.81	1.29	4.30	0.01	60.6%
8	112.8	537.1	2.73	0.90^a^	3.43^a^	1.25^a^	3.10^a^	0.00^a^	64.9%^a^
9	120.5	604.7	2.17	1.04	3.76	1.34	3.91	0.00	72.2%
10	91.6	371.0	3.05	1.00	3.75	1.02	3.76	0.00	65.0%
11	116.8	571.5	2.82	1.07	3.27	1.30	3.49	0.00	61.5%
12	104.4	467.8	3.50	0.39^a^	9.44^a^	1.16^a^	3.69^a^	0.00^a^	66.3%^a^
13	112.1	530.7	3.13	1.14	3.92	1.25	4.49	0.02	75.3%
14	118.1	583.0	3.58	1.09	3.76	1.31	4.08	0.02	57.9%
15	113.0	238.2	3.73	1.04	5.00	1.26	5.20	0.00	68.6%
Avg	115.3 ± 9.0	561.3 ± 75.9	3.15 ± 0.85	1.00 ± 0.00	4.25 ± 1.50	1.28 ± 0.10	3.95 ± 0.71	0.01 ± 0.01	65.9% ± 8.3%
Goal (Minor Deviation)	–	–	<4.00 (<5.00)^b^	CI = 1.00^c^	<4.20^d^	1.11–1.67^e^	<3.63 (<4.85)^e^	<0.15^e^	<65.0% (<83.8%)^e^

a Plan did not pass primary coverage goal.

b Based on departmental standards to prevent overmodulation.

c CI < 1 indicates undercoverage of target; CI > 1 indicates Rx dose spillage beyond target.

d Not considered a hard rule but rather general guidance.^18^

e RTOG0915.^16^

**TABLE 3:** The calculated planning metrics for each of the 10FFF ICARUS plans.

Patient	D_max_ (Gy)	BED_10_ (Gy)	MF	10FFF					
				CI	GI	HI	R50	R105	D2cm
1	112.3	532.4	2.81	0.96^a^	3.74^a^	1.25^a^	3.59^a^	0.01^a^	81.7%^a^
2	121.7	615.6	4.40	1.07	4.14	1.35	4.42	0.00	88.5%
3	124.9	644.4	4.26	1.15	4.15	1.39	4.75	0.01	68.5%
4	102.4	603.4	2.24	0.97^a^	3.78^a^	1.34^a^	3.67^a^	0.01^a^	61.3%^a^
5	115.5	560.5	3.68	1.05	3.40	1.28	3.58	0.00	49.6%
6	122.4	621.7	1.82	1.03	3.51	1.36	3.60	0.01	73.2%
7	115.9	564.0	4.25	1.15	3.64	1.29	4.18	0.01	59.6%
8	119.9	599.1	2.70	1.02	3.29	1.33	3.36	0.00	68.9%
9	118.3	585.1	1.90	1.06	3.70	1.31	3.92	0.01	72.0%
10	92.2	375.3	2.76	1.03	3.72	1.02	3.85	0.00	65.4%
11	116.8	571.9	2.68	1.08	3.24	1.30	3.52	0.00	59.7%
12	116.4	568.0	1.17	1.34	3.70	1.29	4.94	0.08	73.5%
13	118.9	590.5	2.63	1.27	3.62	1.32	4.61	0.08	82.9%
14	120.6	605.4	3.34	1.17	3.54	1.34	4.15	0.02	58.9%
15	115.4	559.3	3.84	1.10	5.21	1.28	5.71	0.01	73.0%
Avg	116.8 ± 7.5	573.1 ± 61.9	2.97 ± 0.18	1.10 ± 0.1	3.76 ± 0.48	1.30 ± 0.08	4.12 ± 0.66	0.02 ± 0.03	69.1 ± 10.4%
Goal (Minor Deviation)	–	–	<4.00 (<5.00)^b^	CI = 1.00^c^	<4.20^d^	1.11–1.67^e^	<3.63 (<4.85)^e^	<0.15^e^	<65.0% (<83.8%)^e^

a Plan did not pass primary coverage goal.

b Based on departmental standards to prevent overmodulation.

c CI < 1 indicates undercoverage of target; CI > 1 indicates Rx dose spillage beyond target.

d Not considered a hard rule but rather general guidance.^18^

e RTOG0915.^16^

The number of MUs/fraction was on average 9456 ± 2551 MU and 8897 ± 2930 MU for the 6FFF and 10FFF plans, respectively. The range was from 3497 to 13 629 MUs over all 30 plans. Note that these values do not necessarily fall within the minimum and maximum MU objectives set during optimization due to the fact that these values are not treated as hard constraints within the system. Nevertheless, these objectives were generally found to be important in achieving plans covering SubGTVs with the desired ultra‐high doses. The modulation factors, shown in Tables 2 and 3, ranged 1.78–4.5 and 1.17–4.4 for the 6FFF and 10FFF plans, respectively. Therefore, while these plans used a relatively high number of MUs in order to deliver the higher target dose, they did not require excessive modulation to avoid exceeding OAR tolerances.

Tables 2 and 3 show the calculated CI, GI, HI, R_50%_, R_105%,_ and D_2cm_ values for each of the 6FFF and 10FFF ICARUS plans, respectively. Plans that did not pass the primary coverage goal are indicated with an asterisk. The CI ranged 0.39–1.19 and 0.96–1.34 for the 6FFF and 10FFF plans, respectively. Plans with a CI value less than one represent plans that did not pass the primary coverage goal or had a lower coverage. The average GI was 4.25 ± 1.50 and 3.76 ± 0.48 for the 6FFF and 10FFF plans, respectively. The average HI was 1.28 ± 0.1 and 1.30 ± 0.08 for the 6FFF and 10FFF plans, respectively. The average R_50%_ values were 3.95 ± 0.71 and 4.12 ± 0.66 for the 6FFF and 10FFF plans, respectively. For all plans, the R_105%_ was less than 0.1, and for 29/30 plans, the D_2cm_ was <83.75%. Note that plans were optimized to meet target coverage and OAR tolerances without necessarily explicitly considering the above metrics, which were used to evaluate plans after the fact. It is possible, if these were used as more strict objectives during optimization, that any of these particular metrics could be improved. The quality of each plan as judged by these different metrics will be dependent on the patient‐specific anatomy (e.g., patient plans with targets more centrally located within the liver did not require as much consideration of lower dose spillage and may tend to have higher R_50_ values). Nevertheless, the above values indicate that these plans are dosimetrically reasonable, based on a variety of widely accepted SBRT planning metrics.

All plans (patients 01–05) measured using the ArcCHECK phantom passed the clinical gamma analysis criteria of 3%/3 mm, 3%/2 mm, and 3%/1 mm with average rates of 99.1% ± 1.0%, 98.5% ± 1.6%, and 95.1% ± 3.8%, respectively. A generally accepted clinical goal is gamma passing at >95% of points, which is satisfied here. This shows that our novel use of treatment planning software can be delivered by the clinical linear accelerator and pass standard patient‐specific QA procedures.

## 4 DISCUSSION

Our previous clinical trial reported on the safety of multisite SBRT in combination with pembrolizumab in patients with metastatic disease. We found that local control was similar in complete or SubGTV tumor SBRT treatments.^7^ These findings led us to explore whether we could feasibly and safely deliver ultra‐high doses of radiation to SubGTV volumes in hopes that this ICARUS strategy can be used on future clinical trials. Other SBRT series investigating dose escalation demonstrate that increasing BED is a promising approach to further improving local control. Li et al demonstrated a BED ≥ 100 was a predictor for 1‐yr local control, with patients receiving BEDs ≥ 100 having a 1‐year local control rate of 96.3% vs 80.6% in patients with BED < 100. Additional studies have demonstrated similar outcomes in other cancers.^10^, ^20^, ^21^ McCammon et al. demonstrated that local control rates can be achieved with even higher BED doses. In primary or metastatic liver tumors who received three fractions of SBRT, patients had local control rates of 89.3%, 59.0%, and 8.1% when they received a BED > 151, a BED between 79 and 151, or a BED < 79, respectively.^9^ Taken together, these findings suggest that using even higher BED may be beneficial in treating patients. Furthermore, preclinical studies demonstrate that higher BED is associated with a greater likelihood of observing an abscopal effect, which is thought to be mediated by an increase in the immune response.^22^ Further studies of radiation therapy at higher BEDs are need to better understand the impact of BED on immune response. Our study demonstrates the feasibility of being able to deliver high BED radiation in order for those studies to be conducted.

The effects of high dose radiation is often seen best in brachytherapy outcomes. Carpenter et al. demonstrated that in extraprostatic prostate cancer treated with trimodality therapy (brachytherapy, external beam radiotherapy, and hormone therapy), higher BED was the only variable that correlated with a higher freedom from biochemical failure. They reported 7‐yr freedom from biochemical failure results of 60% for a BED below 200 and 74% for a BED >200.^23^ Furthermore, high doses of radiation therapy have been shown to be needed for cervical cancer with a brachytherapy boost being standard for locally advanced disease.^24^ Mazzola et. al. used 54 Gy to the whole pelvis with a boost to 66 Gy in 33 fractions to gross disease with an 80% local control rate without severe toxicity as an alternative to brachytherapy (BED_10_ = 79.20 Gy).^25^ On the contrary, a recent UT Southwestern study demonstrated a 70% 2‐yr local control rate after replacing brachytherapy with an SBRT boost; however, there was a progression‐free survival rate of only 47% and the study had to be stopped early due to excess toxicities.^26^ Our method of delivering ultra‐high doses of radiation may be an alternative to brachytherapy by providing a high BED_10_ that could improve local control even more than previous studies, while also respecting normal tissue constraints, particularly in the setting of deep‐seated metastatic tumors that are often not candidates for brachytherapy approaches. Our clinical trial, on which these patients were treated, used an SBRT dose of 45 Gy in three fractions (BED_10_ = 112.5 Gy). In this exploratory dosimetry study, we set out to prescribe to an appreciably higher dose to investigate the upper limit of dosimetric capabilities. Due to the many studies that suggest high BEDs have better patient outcomes, even >220 Gy,^27^ we elected to double the clinical trial dose and theoretically deliver 90 Gy in three fractions, which is equivalent to a BED_10_ = 360 Gy, with BED_10_ maximum doses in the target volume reaching >532 Gy, as seen in Tables 2 and 3.

There is generally a paucity of data relating clinical outcomes to BED on this order; however, models based on retrospective data tend to suggest improved outcomes with increased BED.^28^, ^29^ Future clinical trials utilizing the approach presented herein will be needed to determine ideal dose escalation for improved outcomes with ICARUS treatments. In addition to considering improved outcomes with higher BED, one must also consider the risk of increased toxicity. In general, this work specifically chose to utilize OAR tolerances that were already proven safe in the setting of radiotherapy + immunotherapy. Having demonstrated the capability to deliver such high doses while continuing to respect OAR tolerances, associated OAR toxicities are not expected to increase. However, the out‐of‐field dose, which is poorly modeled in TPSs, will be expected to increase with the increased MUs necessary in these ICARUS plans. The choice to limit MF in this study was to control plan complexity and deliverability, but also to limit the number of MUs and thereby limit this increase in out‐of‐field dose. Based on data from AAPM TG158, we estimate that from these 90 Gy treatments, there will be ~1.0 Gy and ~0.5 Gy at distances of 10 and 20 cm from the field edges, respectively.^30^ While perhaps non‐negligible, the main risks associated with such out‐of‐field dose would be future secondary cancers, which, in the setting of advanced Stage IV patients, is unfortunately less of a concern due to the expected survival timeline, even with hypothetical improvements in outcomes.

The large number of MUs required for these ICARUS plans can either be achieved with the typical 2–4 VMAT arcs by forcing the gantry rotation speed to slow down while maximizing dose rate, or, as in this work, using six to eight partial/full arcs. This alternatively spreads the MU across more arcs, enabling more efficient gantry rotation speed near the maximum 1 revolution per minute (RPM) of the TrueBeam. Additionally, using 6–8 vs 2–4 arcs, partial/full arcs allow for more collimator angles and MLC configurations at any given gantry angle. In doing this, there are greater degrees of freedom within the optimization, while keeping the treatment time approximately the same as when fewer arcs are used (number of MU and dose rate limitations will determine treatment times rather than number of arcs). With gantry rotating near maximum speed of 1 RPM, this results in ~6–8 min per 6–8 arc treatment (average time per arc of 0.99 ± 0.23 min).

Of the three plans that did not pass the primary target coverage, two of the three plans (patients 1 and 12) were due solely to the skin dose constraints. For these patients, the SubGTV was <1.5 mm from the skin contour, which was created using a 5 mm contraction from the patient surface. Utilizing a higher energy (e.g., 10FFF) can improve skin sparing capabilities, which is evidenced by the fact that SubGTV coverage improved from 55.4% to 92.1% and from 41% to 99.7% when switching from 6FFF to 10FFF for these two patients. Patient 8’s 6FFF plan also did not pass the primary target coverage due to both the skin and stomach dose constraints, with the SubGTV < 2.7 mm from the skin contour and <1.2 mm from the stomach contour. Again, the 10FFF beam allowed for better coverage when switching from 6FFF (V_100%_ = 88.0%) to 10FFF (V_100%_ = 97.4%) beams. In addition to better skin sparing, 10FFF beams also have the added benefit of providing double the dose rate compared to 6FFF allowing for faster treatment times, which is of particular importance when delivering these plans which require an average of nearly 10,000 MU/fx; however, there is an added uncertainty of neutron contamination when using higher energy beams. The above characteristics generally associated with lower vs higher energy beams are not necessarily universally true. For example, Patient 4 has improved target coverage for 6FFF vs 10FFF but is not near the patient's skin but rather very central and within the liver. We attribute this to very close proximity to the duodenum, which was the dose limiting OAR in this case. This may imply that for geometries where separation between SubGTV and critical OAR is exceedingly small, lower energy is favorable to minimize lateral travel of secondary particles and associated penumbra broadening. In general, the above considerations for energy choice are guidelines but, in practice, should be individually determined based on each specific case and geometry. An additional note is, while the SubGTVs (based on those used clinically) were uniform contractions, in cases where OAR proximity limits capability to cover targets, one could consider strategically defining the SubGTV as a more distal portion of the GTV. However, the results presented in this work specifically continued to use the SubGTVs implemented and proven to be effective clinically when treated to 45 Gy and did not investigate this alternative approach.

Not only did our ultra‐high dose ICARUS plans meet standard planning goals, we were able to show that our novel use of the TPS resulted in plans that could be delivered by the clinical linear accelerator and pass standard patient QA procedures. All of our treatment plans were delivered to an ArcCHECK phantom and were found to reproduce the planned dose distribution with high fidelity as evidence by gamma passing rates of 95.1% ± 3.8% using a strict 3%/1 mm criteria. Although the plans used a relatively high number of MUs (to be expected for such high prescription doses), the modulation factor for all plans was calculated to be similar to a typical SBRT plan.

While we demonstrate that delivering high doses via external beam SBRT is feasible from a planning perspective, there are additional practical limitations that must be considered before treating patients with such doses. Due to the high‐dose gradients in these ICARUS plans, any uncertainty associated with potential motion may be prohibitive, and motion management and patient immobilization will become even more paramount. Therefore, the presented approach may be more realistic for disease in a region of the body with relatively immobile and/or consistent OARs (e.g., centered within the liver) rather than in locations with less predictable OAR orientations (e.g., near bowel within the abdomen). With the recent development of novel adaptive technologies, real‐time image guidance, and OAR tracking to safely achieve the planned isotoxicity at each treatment,^31^ the field of radiation oncology may begin to push toward ultra‐high dose treatments that focus on delivering the maximal dose to a tumor as governed by the OAR toxicity limitations on any given day. The benefits of such an approach has been recently demonstrated in pancreas SBRT, which had been historically limited by surrounding duodenum/stomach/bowel toxicity concerns,^32^ but for which image‐guided online adaptation has enabled safe delivery of more therapeutic doses. It is also important to note that such high doses may not benefit treatment of all cancer types. Radiosensitive tumors, such as lymphomas, likely do not require the use of high doses; however, patients with more radioresistant tumors may obtain the most benefit from high doses of radiation that were previously limited due to organs at risk. Personalization of radiation therapy may become more common, especially with metastatic patients, where higher doses may be necessary to eradicate more radioresistant tumors.

We should note that patients included in this work were treated on the clinical Phase I trial and therefore were already known to meet OAR constraints when treated to the trial dose of 45 Gy. This essentially selects a subpopulation that did not include patients with targets and anatomy that was unfavorable and perhaps could not be treated on trial. Therefore, the presented results from this ICARUS approach of treating to 90 Gy may not necessarily be applicable to a general patient population, rather applicability will depend on patient‐specific anatomy.

The eponymous Greek mythological character, Icarus, is often remembered for failing to heed the warning of flying too high and close to the sun. It is often forgotten that he was also warned against flying too low and close to the sea. Analogously, while practical considerations may prove delivery of 90 Gy in three fractions to be a goal too high and ambitious, it is also probable that radiation dosing has been too low and cautious in the past, limiting our full potential for tumor control. Our ICARUS planning study demonstrates that it is feasible from a dosimetric prospective to continue to try to find higher doses that can be delivered to tumors to improve tumor control and ultimately patient outcomes. ICARUS's novel planning techniques may be used in future clinical trials to determine the practical implications of our study.

## CONFLICTS OF INTEREST

SJC — Consultancy: RefleXion Medical; Salary: Astellas Pharma (Spouse); Research Support: (all to institution for clinical trials unless noted) Bristol‐Myers Squibb, Merck, EMD Serono, RefleXion.

## Supporting information


**Fig S1**. Example of beam parameters, including gantry, collimator, and couch, rotations for an ICARUS treatment plan.

**Table S1**. OAR dose constraints in NCT02608385.
