# Supplementary material for: Radiation treatment planning study to investigate feasibility of delivering Immunotherapy in Combination with Ablative Radiosurgery to Ultra‐High DoSes (ICARUS)
Source: J Appl Clin Med Phys. 2021 Feb 24;22(3):196–206. doi: 10.1002/acm2.13204 (PMC7984482; doi:10.1002/acm2.13204)
Supplement: Supplementary file 1 — Fig S1. Example of beam parameters, including gantry, collimator, and couch, rotations for an ICARUS treatment plan. [file ACM2-22-196-s002.docx]

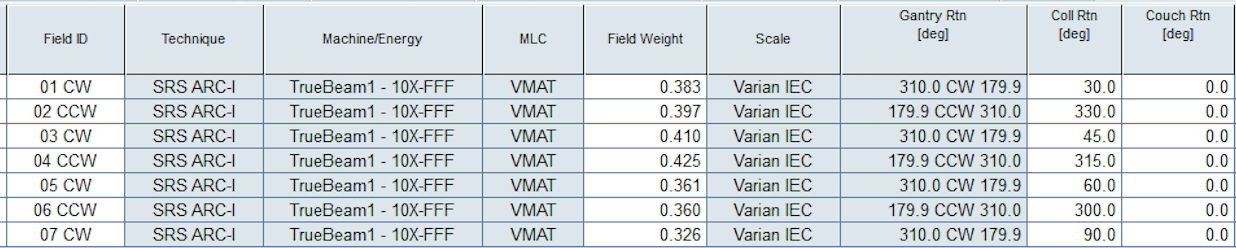


**Supplemental Figure 1:** Example of beam parameters, including gantry, collimator, and couch, rotations for an ICARUS treatment plan
