# Supplementary material for: Radiation treatment planning study to investigate feasibility of delivering Immunotherapy in Combination with Ablative Radiosurgery to Ultra‐High DoSes (ICARUS)
Source: J Appl Clin Med Phys. 2021 Feb 24;22(3):196–206. doi: 10.1002/acm2.13204 (PMC7984482; doi:10.1002/acm2.13204)
Supplement: Supplementary file 2 — Table S1. OAR dose constraints in NCT02608385. [file ACM2-22-196-s001.docx]

| Supplemental Table 1: OAR dose constraints in XXXXXX | | | | |
| --- | --- | --- | --- | --- |
| OAR | **Constraints** | | | |
| Spinal Cord | V22.5Gy < 0.03cc | V13Gy < 1.2cc |  |  |
| Ipsilateral Brachial Plexus | V26Gy < 0.03cc | V22Gy < 3cc |  |  |
| Cauda Equina | V25.5Gy < 0.03cc | V21.9Gy < 5cc |  |  |
| Trachea and Ipsilateral Bronchus | V30Gy < 0.03cc | V25.8Gy < 5cc |  |  |
| Esophagus | V27Gy < 0.03cc | V17.7Gy < 5cc |  |  |
| Heart | V30Gy < 0.03cc | V24Gy < 15cc |  |  |
| Great Vessels | V45Gy < 0.03cc | V 39Gy < 10cc |  |  |
| Skin | V33Gy < 0.03cc | V31Gy < 10cc |  |  |
| Stomach | V30Gy < 0.03cc | V22.5Gy < 10cc |  |  |
| Duodenum | V24Gy < 0.03cc | V15Gy < 10cc |  |  |
| Bowel | V34.5Gy < 0.03cc | V24Gy < 20cc |  |  |
| Rectum | V49.5Gy < 0.03cc | V45Gy < 3.5cc | V27.5Gy < 20cc |  |
| Bladder | V33Gy < 0.03cc | V16.8Gy < 15cc |  |  |
| Ureter | V40Gy < 0.03cc |  |  |  |
| Penile Bulb | V25Gy < 0.03cc |  |  |  |
| Femoral Heads | V25Gy < 10cc |  |  |  |
| Bile Duct | V36Gy < 0.03cc |  |  |  |
| Renal Hilum | V19.5Gy < 15cc |  |  |  |
| Rib | V50Gy < 0.03cc | V40Gy < 5cc |  |  |
| Lung (Total) | V20Gy < 15% | V11Gy < 37% | V10.5Gy < 1500cc | V11.4Gy < 1000cc |
| Ipsilateral Kidney | V12.3Gy < 130cc |  |  |  |
| Total Kidney | V15Gy < 200cc |  |  |  |
| Liver | V17.5Gy < 700cc |  |  |  |
